# Unique Characteristics of Prepubertal Onset Systemic Lupus Erythematosus

**DOI:** 10.1155/2019/9537065

**Published:** 2019-05-27

**Authors:** R. Abdwani, E. Abdalla, I. Al-Zakwani

**Affiliations:** ^1^Pediatric Rheumatology Division, Child Health Department, Sultan Qaboos University Hospital, Muscat, Oman; ^2^Pharmacology and Clinical Pharmacy, College of Medicine & Health Sciences, Sultan Qaboos University, Muscat, Oman

## Abstract

**Objectives:**

The aim of this study was to investigate the influence of age at disease onset on disease expression and outcomes of pediatric systemic lupus erythematosus SLE (pSLE).

**Methods:**

A total of 103 patients with pSLE from Sultan Qaboos University Hospital, Oman, were retrospectively studied. Epidemiological, clinical phenotype, disease severity, serology, treatment, and outcome were compared among the three groups using univariate statistical tests.

**Results:**

The mean disease duration of the cohort was 9.8 ± 4.7 years. The patients were divided into three groups:* pre*pubertal onset (n=39) with mean age at diagnosis of 5.1 ± 2.0 years and pubertal disease onset (n=29) with mean age at diagnosis of 10.8 ± 1.0 years as well as* post*pubertal disease onset (n=35) group with mean age at diagnosis of 15.3 ± 1.6 years. The* pre*pubertal pSLE cohort demonstrates unique characteristics with increased frequency of familial SLE (61%) of which 49% were from first-degree relatives. Similarly, this group had distinctive clinical features, which included increased renal disease in pubertal and* post*pubertal groups, respectively (51%* vs* 23%* vs* 20%;* p*=0.039).* Pre*pubertal, similar to pubertal group, had a higher incidence of cutaneous manifestations than in the* post*pubertal group (74%* vs* 69%* vs* 46%;* p*=0.029). Laboratory features in* pre*pubertal group were distinct with increased frequency of positive anti-cardiolipin antibodies (47%), anti-glycoprotein antibodies (42%), ANCA (62%), and low complement levels (97%) compared to pubertal and* post*pubertal group. The* pre*pubertal group also has the lowest frequency of positive SSA antibodies (18%) and SSB antibodies (5.1%). The overall mean SLEDAI score in pSLE cohort was 15.6 ± 18.5. The mean SLEDAI scores among the groups showed no significance difference (*p*=0.110). The overall SLICC DI ≥1 was 36% with a mean damage score of 0.76 ± 1.38. No significant differences in damage index (SLICC DI ≥1) were noted among the groups.

**Conclusions:**

Distinct clinical features were identified in* pre*pubertal onset pSLE population of Arab ethnicity. Given the high rate of consanguineous marriage and high frequency of familial SLE in this cohort, these manifestations could be explained by higher frequency of genetic factors that influence the disease pathogenesis.

## 1. Introduction

Systemic lupus erythematosus (SLE) is a complex multisystem autoimmune disease characterized by diverse clinical phenotype and course. Currently there are no known distinctive physiological or genetic pathways that can explain the variability in disease phenotypes. However, many factors have been postulated to contribute to the diversity of clinical phenotypes including race, ethnicity, and environmental and socioeconomic factors [1, 2].

Estimates of up to 20% of patients with SLE have the onset of disease before adulthood [3]. More accurate estimates are challenging due to lack of agreement on a clear age limits for the diagnosis of pediatric SLE (pSLE). While the most commonly used age at diagnosis to define pSLE is up to16 years, age ranges of 13 years to 18 years have been used as inclusion criteria for pSLE [3] depending on the practice of the country. In general, the diagnosis of SLE is rare <5 years and uncommon before adolescence [4, 5]. pSLE is in fact the same disease that occurs in adult onset SLE (aSLE); however, substantial differences have been reported [6–13]. Whether those differences are due to variations in biology or genetics has not been fully understood. pSLE has a higher disease severity at presentation, with higher prevalence of lupus nephritis, hematological manifestations, and central nervous system (CNS) involvement as well as constitutional symptoms than aSLE. Comparative studies of adults and children support that pSLE is more often treated with high doses of corticosteroids and immunosuppressive medications than aSLE. pSLE also has higher damage index in ocular, renal, and neuropsychiatric in domains than aSLE. Growth delay, osteoporosis, and negative impact on psychological and physical development, as well as poor treatment compliance, are other issues that need to be addressed in pSLE [6–13].

Previous studies within pSLE population suggest age of disease onset influences the expression of disease in terms of clinical presentation, disease activity, and outcome. Most of those studies were performed in European, Asian, and Latin American pSLE population [14–18]. There have been limited data on the relevance of age in Arab pSLE population. Therefore, the main objective of this study is to compare the effect of pubertal status on pSLE disease expression including clinical phenotype, laboratory findings, treatment, and outcomes among* pre*pubertal, pubertal, and* post*pubertal onset pSLE cohort from Oman.

## 2. Methods

The medical records of patients with the diagnosis of SLE who were followed in the Rheumatology clinics at Sultan Qaboos University Hospital (SQUH) between 2007 and 2017 were reviewed. We included all patients with onset of SLE symptoms ≤18 years and fulfilled the1997 American College of Rheumatology (ACR) revised criteria [19]. Patients were excluded from the study if they did not satisfy any of the SLE classification criteria, had other autoimmune rheumatological disorders, or had missing data at disease onset and those that were lost to follow up at SQUH.

Patients were divided into three groups based on pubertal onset of disease. Puberty in boys is generally achieved between the ages of 9 and 14 years, while in girls it is generally achieved between the ages of 8 and 13 years. The three groups were divided accordingly: the* pre*pubertal group included boys of age of ≤9 years and girls ≤8 years, the pubertal group included boys of age >9 and <14 years and girls >8 and <13 years, and the* post*pubertal group included boys of age ≥14 years and girls ≥13 years. The clinical, laboratory, treatment, and outcomes were compared among each group.

Demographic, clinical presentation and outcome data were retrospectively collected from the hospital electronic information system and analyzed. The demographic characteristics included age at disease onset, gender, disease duration, geographical origin (region of the country), family history of SLE, and degree of consanguinity. Clinical data collected included constitutional symptoms such as fever and weight loss; cutaneous manifestations (malar rash, photosensitivity, discoid rash, and other cutaneous vasculitis), mucosal involvement (oral or nasal ulcers), articular manifestation (arthritis), serositis (pleuritis and pericarditis) renal involvement (proteinuria >0.5 gm/day and cellular casts), central nervous system manifestations (seizures, psychosis, and headache), hematological involvement (leucopenia, lymphopenia, hemolytic anemia, and thrombocytopenia), cardiovascular manifestations (myocarditis, endocarditis, and hypertension), and pulmonary involvement (pneumonitis, shrinking lung syndrome, pulmonary hemorrhage, and pulmonary hypertension). Serological parameters were performed at our local institution including antinuclear antibodies (ANA), which were determined by indirect immunofluorescence using Hep-2 cells as substrate with a cut of >1:80; auto-antibodies including antidouble stranded DNA (anti-dsDNA), antiextractable nuclear antigen (anti-ENA) profile included anti-SSA/Ro, anti-SSB/La, anti-Smith, and anti-RNP, as well as antiphospholipid antibodies were measured qualitatively using enzyme linked immunosorbent assay (ELISA) technique. Auto-antibodies were considered positive if the value was above the cut-off for the laboratory at least in one determination during the follow-up period, except for anti-cardiolipin antibodies, which were considered present if there was two positive occasions, twelve weeks apart. Disease activity at disease onset was assessed using SLE disease activity index (SLEDAI) [20], while assessment of chronic cumulative organ damage was performed using Systemic Lupus International Collaborating Clinics/American College of Rheumatology (SLICC/ACR) damage index [21].

### 2.1. Statistical Analysis

Descriptive statistics were used to describe the data. For categorical variables, frequencies and percentages were reported. Differences between the SLE groups were analyzed using Pearson's *χ*2 tests (or Fisher's exact tests for expected cells <5). For continuous variables, mean and standard deviation were used to present the data while analyses were performed using ordinary least squares (OLS) regression. Survival analyses were analyzed using Kaplan-Meier estimates with differences examined utilizing log-rank test. An a* priori* two-tailed level of significance was set at 0.05. Statistical analyses were conducted using STATA version 13.1 (STATA Corporation, College Station, TX, USA).

## 3. Results

A total of 103 patients with mean disease duration of 9.8 ± 4.73 years were included in the study. The patients were divided into three groups:* pre*pubertal onset (38%; n=39) with a mean age at diagnosis of 5.1 ± 2.0 years; pubertal disease onset (28%; n=29) with mean age at diagnosis of 10.8 ± 1.0 years;* post*pubertal disease onset (34%; n=35) with mean age at diagnosis of 15.3 ± 1.5 years. The demographic characteristics of the patients are described in Table 1. There were 83 (81%) females with an overall female (F):male (M) ratio of 4:1. However, significant differences in female to male ratio were noted across age groups. The F:M ratio increased with increasing age of disease onset from 2:1, 5:1 and 11:1 in* pre*pubertal, pubertal, and* post*pubertal groups, respectively.

pSLE with* pre*pubertal onset had unique characteristics with higher incidence of patients originating from Al-Sharqiya region of Oman (61%), with higher frequency of familial SLE (64%) of which 49% were from first-degree relatives. The* post*pubertal category of pSLE had higher incidence of disease in patients originating from Batinah region of Oman (40%) with lowest incidence of familial SLE (20%). However, the pubertal onset group had a more equal distribution of patients from various regions of Oman.

**Figure 1:**
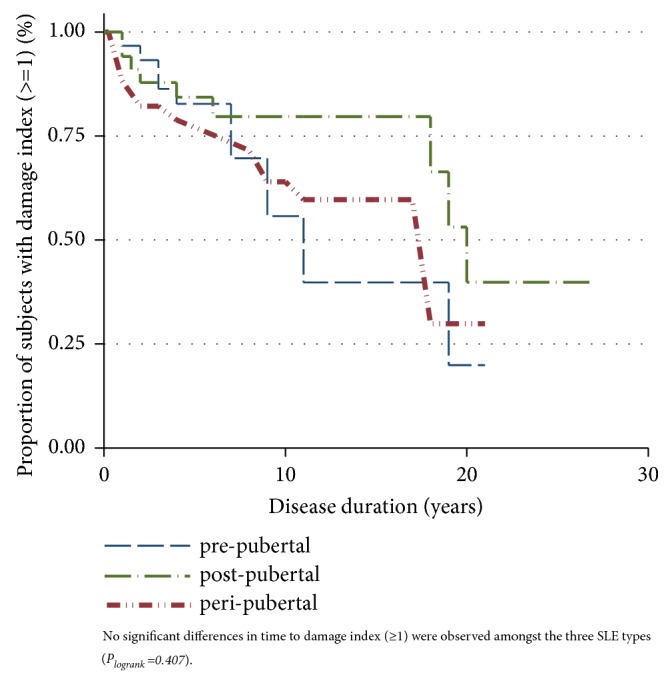
Kaplan-Meir plot of time to damage (index ≥1) stratified by age at disease onset. No significant differences in time to damage index (≥1) were observed among the three SLE types (*P*_*logrank*_*=0.407*).

The most common clinical manifestation of pSLE overall cohort was arthritis (70%) followed by constitutional symptoms (66%), cutaneous manifestation (63%), nephritis (42%), and hematological manifestation (23%).  Table 2 displays the clinical characteristics in each category. The* pre*pubertal pSLE cohort demonstrates unique characteristics which included increased renal disease against pubertal and* post*pubertal groups, respectively (51%* vs* 23%* vs* 20%;* p*=0.039). Of the patients who had renal disease in* pre*pubertal group (n=20), the majority had severe type of nephritis including class III nephritis (25%; n=5) and class IV nephritis (45%; n=9).* Pre*pubertal, similar to pubertal group, had higher incidence of cutaneous manifestation than in* post*pubertal (74%* vs* 69%* vs* 46%;* p*=0.029). However, 51% of cutaneous manifestations in the* pre*pubertal group were urticarial vasculitis, which is a distinguishing feature. In addition,* pre*pubertal group has the lowest hematological involvement compared to the pubertal and* post*pubertal groups, respectively (28%* vs* 66%* vs* 71%;* p*<0.001)). In contrast,* post*pubertal group displayed the least cutaneous, renal, and pulmonary disease. Pubertal group showed no striking distinguishing features in the clinical presentation. The overall mean SLEDAI score in pSLE cohort was 15.6 ± 18.5. The mean SLEDAI score in each group showed no statistical significance difference, while overall SLICC disease index (DI) ≥1 was 36% with an overall mean damage score of 0.76 ± 1.38. No significant difference is SLICC DI ≥1 was noted among the groups as shown in Figure 1.

Table 3 displays the immunological parameters of pSLE cohort. No significant differences were noted in the frequency of ANA and dsDNA in the different age groups. However, the* pre*pubertal category demonstrates unique characteristics with increased frequency of positive anti-cardiolipin antibodies (47%), anti-glycoprotein antibodies (42%), ANCA (62%), and low complement levels (97%) compared to pubertal and* post*pubertal groups. The* pre*pubertal group also has the lowest frequency of positive SSA antibodies (18%) and SSB antibodies (5.1%), while* post*pubertal group had the highest frequency of positive anti-smith antibody (40%). However, pubertal group does not demonstrate any striking laboratory features. The prescribed treatment for pSLE cohort at presentation and during disease course is described in Table 4. All groups were treated similarly with no significant differences noted in the use of corticosteroids or immununosuppressants such as azathioprine, mycophenolate, or cyclophosphamide. However, the* post*pubertal onset pSLE group was treated with rituximab in higher frequency than other groups.

## 4. Discussion

Studies comparing the influence of age on disease presentation and outcome in children with pSLE are scarce [14–18]. The main objective of this study was to investigate the impact of age of disease onset (*pre*pubertal, pubertal, and* post*pubertal) within an Arab pSLE population on disease expression such as differences in clinical presentation, disease activity, serological findings, treatment, and outcomes. The main epidemiological difference between the groups was the F:M gender distribution. The* post*pubertal pSLE has the highest female predominance, which is 5.5 times higher than* peri*pubertal group. There are many theories for the observed female predominance in SLE with growing age, including the influence of female sex hormones in disease pathogenesis most likely observed in* post*pubertal group. Despite the suggested relevance of hormonal factors in the etiology of SLE, a matched cohort study comparing* pre*pubertal with pubertal cSLE demonstrated no important differences in disease features between groups irrespective of pubertal status [17]. Further investigations are needed to study the effects of the hormonal changes that occur during puberty in relations to the mechanisms leading to pSLE.

The* pre*pubertal category has the highest frequency of familial SLE (64%) mainly from Al-Sharqiya region of Oman compared to 30% and 20% in pubertal and postpubertal group, respectively. The role of genetic factors may be relatively more important in the pathogenesis of* pre*pubertal pSLE. In a recent Brazilian study, early onset pSLE (>6 years) patients were associated with complement (C1q, C4, and C2 deficiency) [22]. Recent research has shown that low total C4, C4A, and C4B gene copy number (GCN) were associated with a stronger risk for developing cSLE than adult SLE [22]. Unfortunately, complement function assay was not performed routinely on all the patients although this was strongly suspected as some patients had persistent hypocomplementemia (C3/C4) irrespective of disease activity. However, we identified DNASE1L3 mutation in a number of patients in the* pre*pubertal group with unique clinical characteristics including a high disease activity with variable degree of renal involvement and urticarial vasculitis. Serologically, all patients presented with hypocomplementemia, while most also had positive anti-dsDNA and anti-neutrophil cytoplasmic antibodies [23].

Age of onset of pSLE affects the clinical manifestation of disease. Our study identified* pre*pubertal onset of pSLE with distinct clinical features.* Pre*pubertal onset pSLE had increased cutaneous manifestation and decreased hematological changes with increased tendency of renal disease, while* post*pubertal pSLE had decreased cutaneous and renal disease but increased hematological manifestations. Similarly, a large multicenter study, involving 847 children with pSLE divided into 3 similar groups, identified some distinct clinical and laboratory features in early onset and adolescent groups [16]. The mean SLEDAI score in our cohort, an index of disease activity at disease at onset, showed no statistical difference associated with early onset disease which is similar to other previous studies [16, 18]. However, in another study, infantile SLE were shown to have a more acute disease presentation with a higher disease activity index score (SLEDAI) than in* pre*pubertal (2-10 years) and* post*pubertal (11-16 years) [14].

The overall damage index score demonstrated no significant difference is SLICC DI ≥ 1 as shown in the Kaplan-Meier estimates in our cohort (Figure 1). In the literature, other studies found no relation between the ages of pSLE onset and disease damage as measured by SDI [24, 25], while others showed high SLICC/ACR damage index and death associated with early onset SLE (<5 years) [26]. Our findings are different from the results of aSLE population for whom increasing ages are correlated with increasing damage score [27–29].

In our cohort,* pre*pubertal onset pSLE had significantly higher frequency of positive ANCA, anti-cardiolipin antibody, anti-glycoprotein antibody, and higher frequency of lower complement levels. This group also displayed lower SSA, SSB, and antinucleosome antibodies, while* post*pubertal displayed higher anti-smith antibodies. Ethnic variations in the frequency of auto-antibodies with pSLE have been described previously [5, 17]. In two previous studies of Italian and Brazilian pSLE population, no significant differences in auto-antibody profile were identified between the three groups of pSLE population [15, 16]. The* pre*pubertal group in our cohort displayed a higher frequency of familial SLE, in which DNASE1L3 gene mutation was identified in 13 patients who were tested and had characteristic features of early age of onset and positive ANCA [23]. Similarly, high frequency of positive anticardiolipin positivity may be explained by infectious process that may result in transient and nonpathogenic rise in antibody titer which may occur more commonly in younger children. In general, antiphospholipid antibody related thrombosis is rare in children and none of our patients developed any thrombotic events.

Comparative studies of adults and children support that pSLE is more often treated with high doses of corticosteroids and immunosuppressive medications than aSLE [15]. However, comparative studies within pSLE reveal no major difference in treatment frequencies and strategies according to various ages of onset groups [7, 9]. Similarly, our cohort of patients did not reveal significant differences. All patients were treated with prednisolone, hydroxychloroquine, and various immunosuppressive agents such as azathioprine, mycophenolate, and cyclophosphamide in comparable frequencies. However, rituximab was more frequently used in postpubertal group. Indications for using rituximab in the* post*pubertal group (n=19) were refractory lupus nephritis (63%), CNS vasculitis (13%), persistent arthritis (13%), autoimmune hemolytic anemia (6.3%), and mesenteric vasculitis (6.3%).

This study is not without limitations. One of the major limitations is the relatively small sample size. However, given the uncommon nature of illness, the sample size is considered reasonable to conduct the study. Furthermore, the retrospective nature of the study is another limitation. As the tanner staging was not included routinely in the medical records, we were not able to evaluate the effect of disease on pubertal delay as a reflection of damage index. Despite these limitations, we had no significant missing data to affect the findings of the study. Additionally, we had equal number of patients in each group, despite the rare occurrence of disease in children >5 years of age, to make the groups comparable. Finally, the cohort was a true representation of an Omani cohort of Arab ethnicity.

## 5. Conclusion

Our study demonstrates unique disease expression in* pre*pubertal onset pSLE related to epidemiological, clinical, and laboratory features.* Pre*pubertal onset pSLE had a higher frequency of familial SLE associated and lower F:M ratio than other groups. Clinically,* pre*pubertal onset SLE had high renal and cutaneous manifestations of SLE. The laboratory features demonstrated higher anti-cardiolipin antibody, anti-glycoprotein antibody, anti-neutrophic cytoplasmic antibody, and lower complements than the other groups. Given the high rate of consanguineous marriage in Oman and a high frequency of familial SLE in this cohort (39.8%), these manifestations could be explained by a higher frequency of genetic factors that influence the disease pathogenesis [30]. Further genetic studies are warranted to identify additional genetic loci and to enhance our understanding of the pathogenesis of SLE.

## Figures and Tables

**Table 1:** Characteristics and outcomes of systemic lupus erythematosus (SLE) patients according to age at onset of disease.

*Characteristic*, n (%) unless specified otherwise	Pre-pubertal	Peri-pubertal	Post-pubertal	*P*-value
	n=39	n=29	n=35	
Age at disease onset (years)				
Girls	≤ 8 (69%; n=27)	> 8 and <13	≥ 13(91%; n=32)	0.056
		(83%; n=24)		
Boys	≤ 9 (31%; n=12)	>9 and <14	≥14 (8.6%; n=3)	
		(17%; n=5)		

Age, mean ± SD, years	5.12 ± 1.98	10.8 ± 0.99	15.3 ± 1.59	<0.001

Sex ratio (F: M)	1: 2 (27:12)	1:5 (24:5)	1: 11 (32:3)	0.056

Region				<0.001
Sharqiya	61%; n=24	24%; n=7	14%; n=5	
Batina	15%; n=6	24%; n=7	40%; n=14	
Muscat	5.1%; n=2	28%; n=8	29%; n=10	
Dhakhilia	5.1%; n=2	21%; n=6	11%; n=4	
Dhahira	0	3.4%; n=1	2.9%; n=1	
Dhofar	13%; n=5	0	0	
Musandam	0	0	2.9%; n=1	

Family history of SLE	25 (64%)	9 (31%)	7 (20%)	<0.001

1^st^ degree	19 (49%)	4 (14%(	5 (14%)	0.001

DI ≥1	15 (39%)	12 (41%)	10 (29%)	0.520

Mortality	3 (7.7%)	1 (3.4%)	1 (2.9%)	0.626

SD: standard deviation; F: female; M: male; DI: damage index.

**Table 2:** Clinical manifestations of systemic lups erythematosus (SLE) patients according to age at onset of disease.

*Characteristic*s	Pre-pubertal	Peri-pubertal	Post-pubertal	*P*-value
n (%)	n=39	n=29	n= 35	
Cutaneous	74.4% (29)	69%(20)	45.7%(16)	0.029
	UV (53.8%)	alopecia (27.6%)	alopecia (20%)	

Nephritis	20 (51%)	10 (34%)	8 (23%)	0.039

Hematological	11 (28%)	19 (66%)	25 (71%)	<0.001

Arthritis	30 (77%)	19 (66%)	21 (60%)	0.281

Neurological	4 (10%)	6 (21%)	5 (14%)	0.500

Pulmonary	6 (15%)	4 (14%)	0	0.031

Serositis	8 (21%)	7 (24%)	3 (8.6%)	0.201

Constitutional symptoms	28 (72%)	21 (72%)	17 (49%)	0.063

SLEDAI (severe activity)	24 (61%)	18 (62%)	14 (40%)	0.110

SLDEDAI: Systemic Lupus Erythematosus Disease Activity Index.

**Table 3:** Investigations of systemic lups erythematosus (SLE) patients according to age at onset of disease.

Investigation type, n (%)	Pre-pubertal	Peri-pubertal	Post-pubertal	*P*-value
	n=39	n=29	n=35	
Anti smith	3 (7.7%)	7 (24%)	14 (40%)	0.003
Anti SSA	7 (18%)	12 (41%)	15 (43%)	0.040
Anti SSB	2 (5.1%)	5 (17%)	11 (31%)	0.004
Anti nucleosome*∗*	3 (7.7%)	5 (17%)	11 (37%)	0.011
Anti cardiolipin	17 (44%)	10 (34%)	5 (14%)	0.022
Anti glycoprotein	15 (38%)	8 (28%)	4 (11%)	0.025
ANCA	24 (62%)	11 (38%)	4 (11%)	<0.001
Low C3	38 (97%)	23 (79%)	27 (77%)	0.016

*∗*Percentage of antinucleosome among postpubertal is out of 30 as 5 were missing and not available.

**Table 4:** Medications of systemic lupus erythematosus (SLE) patients according to age at onset of disease.

Medications, n (%)	Pre-pubertal	Peri-pubertal	Post-pubertal	*P*-value
	n=39	n=29	n=35	
NSAIDS	20 (51%)	12 (41%)	11 (31%)	0.224
Hydroxychloroquin (HCQ))	38 (100%)	28 (97%)	34 (97%)	0.851
Prednisolone	39 (100%)	29 (100%)	28 (80%)	0.001
IVMP	20 (51%)	19 (66%)	27 (77%)	0.067
Azathioprine	27 (69%)	22 (76%)	24 (69%)	0.783
Methotrexate	10 (26%)	0	12 (34%)	0.001
Mycophenolate mofetil	25 (64%)	18 (62%)	25 (71%)	0.697
Cyclophosphamide	20 (51%)	15 (52%)	20 (57%)	0.861
Rituximab	6 (15%)	5 (17%)	15 (43%)	0.013
Immunoglobulin	3 (7.7%)	3 (10%)	7 (20%)	0.256

NSAIDs: nonsteroidal anti-inflammatory drugs; IVMP: intravenous methylprednisolone.

HCQ was missing in 1 patient under prepubertal.

## Data Availability

The data used to support the findings of this study are available from the corresponding author upon request.

## Additional Points

There is sparse data emanating from Arab world on pediatric onset SLE. The authors believe that it would be important to share the results of their study as there is heterogeneity in disease epidemiology and presentation around the world. By sharing experiences, they would have better understanding of disease pathogenesis.
